# Prevalence and molecular characteristics of drug-resistant *Mycobacterium tuberculosis* in Hainan, China: from 2014 to 2019

**DOI:** 10.1186/s12866-021-02246-7

**Published:** 2021-06-19

**Authors:** Lin Liu, Xiujuan Zhao, Xingyong Wu, Sijing Li, Biao Liu, Mamy Jayne Nelly Rajaofera, Yingfei Zeng, Sufang Dong, Zheng Bei, Hua Pei, Qianfeng Xia

**Affiliations:** 1 NHC Key Laboratory of Control of Tropical diseases, Key Laboratory of Tropical Translational Medicine of Ministry of Education, School of Tropical Medicine and Laboratory Medicine, Hainan Medical University, Haikou, 571199 China; 2 Public Health School, Hainan Medical University, Haikou, 571199 Hainan China; 3 Hainan Province cadre sanatorium, Hainan Province Geriatric Hospital, Haikou, 571100 China; 4 Department of Clinical Laboratory, The Second Affiliated Hospital, Hainan Medical University, Haikou, 570311 China

**Keywords:** *Mycobacterium tuberculosis*, DNA microarray, Drug sensitivity test, Molecular-characteristics, Drug-resistant rate

## Abstract

**Background:**

The emergence of antimicrobial resistance against *Mycobacterium tuberculosis* (*M. tuberculosis*) has become the major concern in global tuberculosis control due to its limited therapy options and high mortality. However, the clinical and molecular characteristics of drug-resistant strains vary in different geographical areas. Hainan Island located in southern China, is a high drug-resistant tuberculosis burden area. This study aimed to determine the dynamic changes of drug-resistance patterns and drug-related gene mutation types of *M. tuberculosis* in Hainan from 2014 to 2019.

**Results:**

A total of 1484 culture-confirmed *M. tuberculosis* were included in this study. It was found that the proportions of drug resistance to isoniazid and rifampin were 31.3 and 31.1% respectively. Overall the proportion of multidrug resistant *M. tuberculosis* was 24.9%. Multivariate logistic regression analysis showed that age and the treatment history were independent influencing factors of drug resistant tuberculosis. The proportions of drug-resistant tuberculosis in retreatment patients were considerably higher than those in new patients. The most common mutation types of isoniazid were Ser315 → Thr (66.3%), and the most common mutation types of rifampin were Ser531 → Leu (41.5%).

**Conclusions:**

Our data suggests that the prevalence of drug resistant TB remains high in Hainan, and the risks for developing drug resistance with diversified mutation types increased significantly in retreatment patients. These results contribute to the knowledge of the prevalence of drug resistance in Hainan Province and expand the molecular characteristics of drug resistance in China simultaneously.

## Background

*Mycobacterium tuberculosis* (*M. tuberculosis*) is a main pathogen of tuberculosis (TB)*.* It can affect almost all human organs, especially the lung [1–3]. The emergence of Drug-resistant tuberculosis (DR-TB), particularly multidrug-resistant *tuberculosis* (MDR-TB), caused by *M. tuberculosis* strains resistant to at least isoniazid and rifampin) and extensively drug-resistant tuberculosis (XDR-TB, MDR-TB with additional resistance to a second-line fluoroquinolone and injectable drug), has been identified as one of the major obstacles to effective TB control in many countries [4, 5]. It was estimated that 10.0 million (range, 8.9–11.0 million) people fell ill with TB in 2019, according to a newest report from the WHO, and there were an estimated 465,000 (range, 400,000–535,000) incident cases of rifampicin-resistant TB, of which 78% were MDR-TB.

DR-TB is usually associated with delayed diagnosis, prolonged or ineffective treatment or direct transmission of drug-resistant strains from one individual to another [6, 7]. The magnitude and pattern of drug resistance varied greatly with the region because of the huge size of the country, the diverse population density, and the unbalanced economic development in China [8]. Hainan is the southernmost island in China. Separated from the mainland China by the Qiongzhou Strait, the population mobility of Hainan Province is lower than that of other Provinces. *M. tuberculosis* of Hainan Province may demonstrate a unique genetic evolution due to the unique geographical location. Unfortunately, thus far, the true magnitude of DR-TB of Hainan Province was not well described to date and should be explored to facilitate control of the TB epidemic in this region and throughout China.

To better understand the clinical and molecular characteristics of *M. tuberculosis* isolates, we analyzed all strains collected from TB inpatients admitted to the Second Affiliated Hospital of Hainan Medical University from 2014 to 2019. Clinical information, drug-resistant phenotypes and drug-resistance associated mutation types were compared. This study was to evaluate the clinical characteristics and changes in molecular epidemiology of DR-TB.

## Results

### Demographic and clinical characteristics

**Fig. 1:**
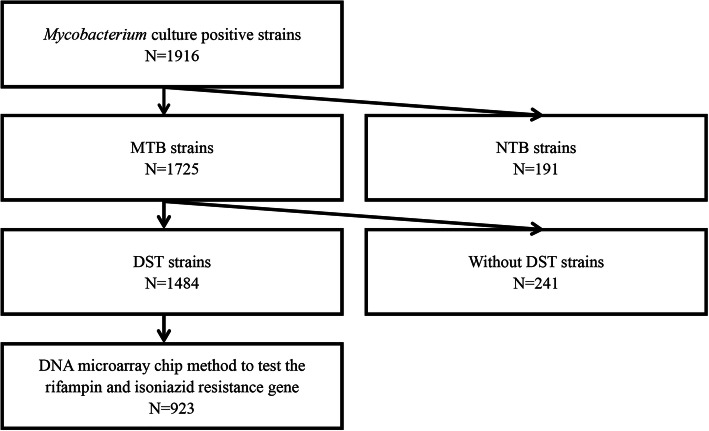
Inclusion and Exclusion of the study objects. DST: drug susceptibility test

We analyzed the demographic and clinical information of culture-confirmed *M. tuberculosis* in Hainan from January 1, 2014 to December 31, 2019. A total of 1484 *M. tuberculosis* strains were included, and 923 of which were tested for rifampin and isoniazid drug-resistance gene mutation types by DNA microarray (Fig. 1).

Totally 223, 171, 107, 190, 331 and 462 isolates were tested for drug sensitivity from 2014 to 2019, respectively. The mean age of patients was 48 ± 17.5 (range: 1–95) years old and the gender ratio was 4.6. Gender and contact history showed no statistically significant change using the Chi-square test for trends (*P* > 0.05) while, age and treatment history changed significantly (*P* < 0.05). Compared with results in 2014, the proportion of patients aged between 25 to 64 was significantly higher in 2015 (*P* = 0.028), the proportion of patients aged over 44 was significantly higher in 2019 (*P* = 0.032), and the proportion of retreatment cases was significantly higher in 2016 (*P* < 0.001). What is worth mentioning is that, the proportion of new cases in 2019 (61.3%) was higher than that in 2014 (55.6%) (Table 1).

**Table 1:** Characteristics of study population from 2014 to 2019

Characteristics	Total	2014	2015	2016	2017	2018	2019	χ^2^	*P*
	n (%)	n (%)	n (%)	n (%)	n (%)	n (%)	n (%)		
Gender									
Male	1217 (82.0)	181 (81.2)	147 (86.0)	84 (78.5)	157 (82.6)	262 (79.2)	386 (83.5)	5.433	0.365
Female	267 (18.0)	42 (18.8)	24 (14.0)	23 (21.5)	33 (17.4)	69 (20.8)	76 (16.5)		
Age group (years)									
~ 25	205 (13.8)	42 (18.8)	18 (10.5)	17 (15.9)	34 (17.9)	43 (13.0)	51 (11.0)	25.692	0.041
~ 44	375 (25.3)	55 (24.7)	55 (32.2)	26 (24.3)	43 (22.6)	88 (26.6)	108 (23.4)		
~ 64	635 (42.8)	88 (39.5)	78 (45.6)	48 (44.9)	81 (42.6)	130 (39.3)	210 (45.5)		
> 64	269 (18.1)	38 (17.0)	20 (11.7)	16 (15.0)	32 (16.8)	70 (21.1)	93 (20.1)		
Treatment history									
New cases	825 (55.6)	125 (56.1)	88 (51.5)	39 (36.4)	94 (49.5)	196 (59.2)	283 (61.3)	27.727	< 0.001
Retreatment	659 (44.4)	98 (43.9)	83 (48.5)	68 (63.6)	96 (50.5)	135 (40.8)	179 (38.7)		
cases									
Contact history									
No	1313 (88.5)	206 (92.4)	154 (90.1)	90 (84.1)	167 (87.9)	285 (86.1)	411 (89.0)	7.745	0.171
Yes	171 (11.5)	17 (7.6)	117 (9.9)	17 (15.9)	23 (12.1)	46 (13.9)	51 (11.0)		
Total	1484 (100.0)	223 (15.0)	171 (11.5)	107 (7.2)	190 (12.8)	331 (22.3)	462 (31.1)		

### Drug susceptibility patterns

Changes of drug-resistance pattern of *M. tuberculosis* from 2014 to 2019 were shown in Table 2. Analysis of the 1484 culture-confirmed TB cases showed that the isoniazid resistant TB rate was 31.3%, the rifampin resistant TB rate was 31.1%, the MDR-TB rate was 24.9%, and the XDR-TB rate was 2.2%. In addition, any-drug-resistant TB accounted for 25.8% of new cases and 67.5% of retreatment cases. The isoniazid resistant TB accounted for 14.8% of new cases and 52.0% of retreatment cases. The rifampin resistant TB accounted for 11.4% of new cases and 55.7% of retreatment cases. MDR-TB accounted for 8.6% of new cases and 45.2% of retreatment cases. XDR-TB accounted for 0.4% of new cases and 4.6% of retreatment cases. For the entire study cohort (1484 cases), the longitudinal changes in overall percentage of rifampin (RIF) resistance, kanamycin (KAR) resistance and protionamide (PTO) resistance overtime showed a statistically significant increase using the Chi-square test for trends (Table 2).

**Table 2:** Evaluation and comparison of drug resistance rate of anti-*tuberculosis* drugs in 2014–2019

Category	2014 n (%)	2015 n (%)	2016 n (%)	2017 n (%)	2018 n (%)	2019 n (%)	Total n (%)	χ^2^	*P*
**All TB cases**									
Any drug-resistance	95 (42.6)	85 (49.7)	60 (56.1)	96 (50.5)	127 (38.4)	195 (42.2)	658 (44.3)	16.820	0.005
INH	71 (31.8)	67 (39.2)	42 (39.3)	68 (35.8)	85 (25.7)	132 (28.6)	465 (31.3)	16.349	0.006
RIF	60 (26.9)	63 (36.8)	48 (44.9)	68 (35.8)	86 (26.0)	136 (29.4)	461 (31.1)	20.521	0.001
EMB	23 (10.3)	12 (7.0)	20 (18.7)	26 (13.7)	26 (7.9)	41 (8.9)	148 (10.0)	15.941	0.007
STR	48 (21.5)	39 (22.8)	24 (22.4)	50 (26.3)	56 (16.9)	98 (21.2)	315 (21.2)	6.977	0.222
MDR	54 (24.2)	50 (29.2)	38 (35.5)	53 (27.9)	63 (19.0)	111 (24.0)	369 (24.9)	15.430	0.009
Any second-line drug resistance	42 (18.8)	43 (25.1)	34 (31.8)	46 (24.2)	47 (14.2)	96 (20.8)	308 (20.8)	20.435	0.001
CPM	2 (0.9)	3 (1.8)	3 (2.8)	7 (3.7)	6 (1.8)	21 (4.5)	42 (2.8)	10.443	0.064
KAR	6 (2.7)	5 (2.9)	7 (6.5)	13 (6.8)	6 (1.8)	14 (3.0)	51 (3.4)	13.119	0.022
OFX	38 (17.0)	41 (24.0)	33 (30.8)	42 (22.1)	43 (13.0)	76 (16.5)	273 (18.4)	24.208	< 0.001
PTO	1 (0.4)	1 (0.6)	0 (0.0)	0 (0.0)	0 (0.0)	18 (3.9)	20 (1.3)	34.796	< 0.001
XDR	4 (1.8)	3 (1.8)	5 (4.7)	10 (5.3)	4 (1.2)	7 (1.5)	33 (2.2)	11.580	0.041
**New cases**									
Any drug-resistance	35 (28.0)	22 (25.0)	13 (33.3)	29 (30.9)	47 (24.0)	67 (23.7)	213 (25.8)	3.759	0.585
INH	22 (17.6)	14 (15.9)	6 (15.4)	17 (18.1)	27 (13.8)	36 (12.7)	122 (14.8)	2.813	0.729
RIF	15 (12.0)	8 (9.1)	7 (17.9)	13 (13.8)	23 (11.7)	28 (9.9)	94 (11.4)	3.181	0.672
EMB	6 (4.8)	2 (2.3)	1 (2.6)	4 (4.3)	6 (3.1)	11 (3.9)	30 (3.6)	1.462	0.917
STR	17 (13.6)	9 (10.2)	6 (15.4)	13 (13.8)	25 (12.8)	31 (11.0)	101 (12.2)	1.604	0.901
MDR	14 (11.2)	4 (4.5)	5 (12.8)	8 (8.5)	15 (7.7)	25 (8.8)	71 (8.6)	4.210	0.520
Any second-line drug resistance	13 (10.4)	7 (8.0)	7 (17.9)	11 (11.7)	12 (6.1)	26 (9.2)	76 (9.2)	6.397	0.270
CPM	1 (0.8)	1 (1.1)	0 (0.0)	3 (3.2)	3 (1.5)	7 (2.5)	15 (1.8)	4.133	0.530
KAR	2 (1.6)	1 (1.1)	0 (0.0)	4 (4.3)	1 (0.5)	6 (2.1)	14 (1.7)	6.696	0.244
OFX	10 (8.0)	7 (8.0)	7 (17.9)	8 (8.5)	10 (5.1)	17 (6.0)	59 (7.2)	7.388	0.193
PTO	0 (0.0)	0 (0.0)	0 (0.0)	0 (0.0)	0 (0.0)	2 (0.7)	2 (0.2)	4.289	0.509
XDR	0 (0.0)	1 (1.1)	0 (0.0)	1 (1.1)	0 (0.0)	1 (0.4)	3 (0.4)	4.383	0.496
**Retreatment cases**									
Any drug-resistance	60 (61.2)	63 (75.9)	47 (69.1)	67 (69.8)	80 (59.3)	128 (71.5)	445 (67.5)	10.236	0.069
INH	49 (50.0)	53 (63.9)	36 (52.9)	51 (53.1)	58 (43.0)	96 (53.6)	343 (52.0)	9.512	0.090
RIF	45 (45.9)	55 (66.3)	41 (60.3)	55 (57.3)	63 (46.7)	108 (60.3)	367 (55.7)	14.257	0.014
EMB	17 (17.3)	10 (12.0)	19 (27.9)	22 (22.9)	20 (14.8)	30 (16.8)	118 (17.9)	9.294	0.098
STR	31 (31.6)	30 (36.1)	18 (26.5)	37 (38.5)	31 (23.0)	67 (37.4)	214 (32.5)	10.845	0.055
MDR	40 (40.8)	46 (55.4)	33 (48.5)	45 (46.9)	48 (35.6)	86 (48.0)	298 (45.2)	10.328	0.066
Any second-line drug resistance	29 (29.6)	36 (43.4)	27 (39.7)	35 (36.5)	35 (25.9)	70 (39.1)	232 (35.2)	10.741	0.057
CPM	1 (1.0)	2 (2.4)	3 (4.4)	4 (4.2)	3 (2.2)	14 (7.8)	27 (4.1)	10.541	0.061
KAR	4 (4.1)	4 (4.8)	7 (10.3)	9 (9.4)	5 (3.7)	8 (4.5)	37 (5.6)	6.544	0.257
OFX	28 (28.6)	34 (41.0)	26 (38.2)	34 (35.4)	33 (24.4)	59 (33.0)	214 (32.5)	8.806	0.117
PTO	1 (1.0)	1 (1.2)	0 (0.0)	0 (0.0)	0 (0.0)	16 (8.9)	18 (2.7)	35.332	< 0.001
XDR	4 (4.1)	2 (2.4)	5 (7.4)	9 (9.4)	4 (3.0)	6 (3.4)	30 (4.6)	7.670	0.175

Abbreviation: INH, isoniazid; RIF, rifampin; EMB, ethambutol; STR, streptomycin; first-line drug resistance, including isoniazid, rifampin ethambutol and streptomycin; MDR, multi-drug resistant; CPM, capreomycin; KAR, kanamycin; OFX, ofloxacin; PTO, protionamide; XDR, extensively drug-resistant

**Fig. 2:**
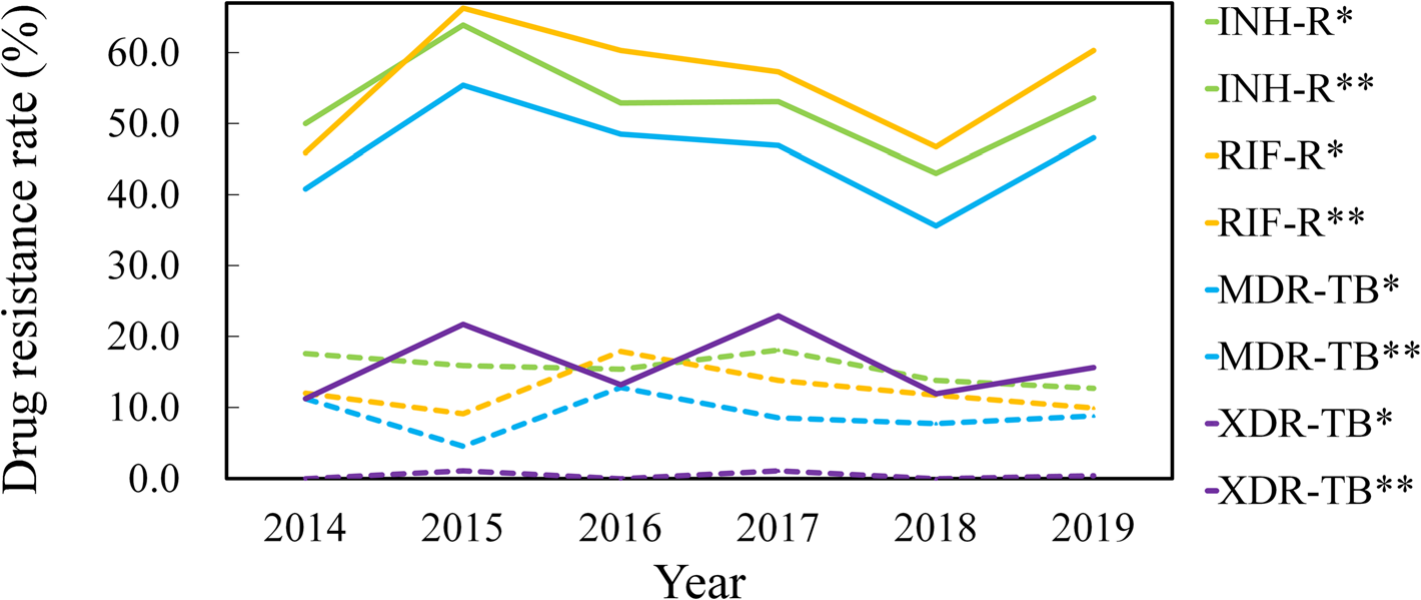
Trends of different drug-resistance patterns among 1484 culture-confirmed TB cases in Hainan, 2014 to 2019. In new cases, for INH resistance (χ^2^ = 2.813, *P* = 0.729); for RIF resistance (χ^2^ = 3.181, *P* = 0.672); for MDR-TB (χ^2^ = 4.210, *P* = 0.520); for XDR-TB (χ^2^ = 4.383, *P* = 0.496). In retreatment cases, for INH resistance (χ^2^ = 9.512, *P* = 0.090); for RIF resistance (χ^2^ = 14.257, *P* = 0.014); for MDR-TB (χ^2^ = 10.328, *P* = 0.066); for XDR-TB (χ^2^ = 7.670, *P* = 0.175). Note: *new cases, **retreatment cases. Abbreviation: INH-R, isoniazid resistance; RIF-R, rifampin resistance; MDR-TB, multidrug resistant tuberculosis; XDR-TB, extensively drug resistant tuberculosis

To have a better understanding of the epidemic trends in tuberculosis cases with different treatment histories, we explored the changes of drug resistance rate in newly treated patients and retreatment patients respectively. There was no statistical significance in changes of the drug resistance rate in the new TB cases (*P* > 0.05), while in retreatment TB cases, RIF resistance increased at an annual rate of 0.05% (Chi-square test for trends: χ^2^ = 14.257, *P* = 0.014), PTO resistance increased at an annual rate of0.18% (Chi-square test for trends: χ^2^ = 35.332, *P* < 0.001) (Fig. 2).

### Factors associated with drug-resistance TB

Both univariate and multivariate analysis showed that age and treatment history were influencing factors for TB patients being resistant to any anti-TB drug, multidrug-resistant or extensive drug-resistant (*P* < 0.05) (Table 3). The risk of drug-resistance was associated with lower age. For those aged > 64 years, the odd ratio to any drug-resistance, MDR and XDR were, respectively, 0.602 (95% confidence interval (CI): 0.397, 0.913), 0.277 (95%CI: 0.157, 0.486) and 0.287 (95%CI: 0.117, 0.702) times of those under 25 years old (*P* = 0.017 for any tested-drug; *P* < 0.001 for MDR; *P* = 0.006 for XDR). Furthermore, the risk of drug-resistant in retreatment cases was significantly higher than that in new cases, and their risk of resistance to any tested-drug, multi-drug-resistant, extensive drug-resistant were respectively, 5.958(95%CI: 4.738, 7.492), 12.753(95%CI: 9.349, 17.397) and 16.498(95%CI: 9.816, 27.728) times higher than that in new cases (*P* < 0.001 for any tested-drug, *P* < 0.001 for multi-drug-resistant, *P* < 0.001 for extensive drug-resistant). Compared to people with no exposure history, those who have exposure history may have higher drug resistance rates, and their risk of resistance to any detected drugs were 1.428 (95% CI: 1.000, 2.039) times higher than that of the people without contact history (*P* = 0.050).

**Table 3:** Factors associated with drug-resistance TB

Factors	Any drug resistant TB (*n* = 658)	MDR-TB (*n* = 369)	XDR-TB (*n* = 123)	Pan-susceptible TB (*n* = 826)	Any drug resistant TB VS Pan-susceptible TB				MDR-TB VS Pan-susceptible TB				XDR-TB VS Pan-susceptible TB			
					OR (95%CI)	*P*	Adjusted OR (95%CI)	*P*	OR (95%CI)	*P*	Adjusted OR (95%CI)	*P*	OR (95%CI)	*P*	Adjusted OR (95%CI)	*P*
**Gender**																
Male	534 (81.2)	299 (81.0)	97 (78.9)	683 (82.7)	Reference				Reference				Reference			
Female	124 (18.8)	70 (19.0)	26 (21.1)	143 (17.3)	0.83 (0.614,1.121)	0.225			0.82 (0.555,1.209)	0.316			0.673 (0.387,1.172)	0.162		
**Age (years)**																
~ 25	84 (12.8)	52 (14.1)	15 (12.2)	121 (14.6)	Reference		Reference		Reference		Reference		Reference		Reference	
~ 44	173 (26.3)	100 (27.1)	31 (25.2)	202 (24.5)	1.08 (0.737,1.582)	0.694	1.052 (0.720,1.536)	0.795	0.955 (0.588,1.550)	0.852	0.917 (0.568,1.482)	0.725	1.129 (0.540,2.363)	0.747	1.062 (0.511,2.205)	0.873
~ 64	311 (47.3)	183 (49.6)	67 (54.5)	324 (39.2)	1.079 (0.753,1.544)	0.679	1.039 (0.730,1.479)	0.833	0.941 (0.598,1.480)	0.791	0.886 (0.568,1.328)	0.593	1.329 (0.674,2.622)	0.412	1.227 (0.629,2.390)	0.549
> 64	90 (13.7)	34 (9.2)	10 (8.1)	179 (21.7)	0.626 (0.410,0.954)	0.030	0.602 (0.397,0.913)	0.017	0.3 (0.169,0.533)	< 0.001	0.277 (0.157,0.486)	< 0.001	0.317 (0.128,0.786)	0.013	0.287 (0.117,0.702)	0.006
**Treatment history**																
New cases	213 (32.4)	71 (19.2)	19 (15.4)	612 (74.1)	Reference		Reference		Reference		Reference		Reference		Reference	
Retreatment cases	445 (67.6)	298 (80.8)	104 (84.6)	214 (25.9)	5.992 (4.763,7.538)	< 0.001	5.958 (4.738,7.492)	< 0.001	12.777 (9.356,17.448)	< 0.001	12.753 (9.349,17.397)	< 0.001	16.778 (9.959,28.268)	< 0.001	16.498 (9.816,27.728)	< 0.001
**Contact history**																
No	565 (85.9)	317 (85.9)	108 (87.8)	748 (90.6)	Reference		Reference		Reference				Reference			
Yes	93 (14.1)	52 (14.1)	15 (12.2)	78 (9.4)	1.408 (0.985,2.014)	0.060	1.428 (1.000,2.039)	0.050	1.247 (0.794,1.960)	0.338			1.049 (0.532,2.069)	0.889		

Note: * indicates that the difference is statistically significant compared with 2014

### Detection of drug resistance-associated mutations by DNA microarray

The gene mutation profile of *M. tuberculosis* resistant strains was further investigated. A total of 923 strains were tested for resistance gene mutations by DNA microarray and the mutation sites were shown in Table 4.

**Table 4:** Evolution of drug resistance mutation sites of *Mycobacterium tuberculosis* to first-line anti *tuberculosis* drugs isoniazid and rifampin in 2014–2019

Drug	Locus	Nucleic acid change	Codon mutation	2014 n (%)	2015 n (%)	2016 n (%)	2017 n (%)	2018 n (%)	2019 n (%)	Total
INH	*KatG*	AGC → AAC	*Ser*315*Asn*	4 (10.0)	0 (0.0) ^*^	0 (0.0) ^*^	1 (2.7)	3 (6.3)	5 (5.2)	13 (4.5)
		AGC → ACC	*Ser*315*Thr*	30 (75.0)	5 (11.1)	10 (47.6)	34 (91.9)	40 (83.3)	72 (74.2)	191 (66.3)
	*inhA*	C → T	*C*(−15) → *T*	0 (0.0)	0 (0.0)	1 (4.8)	2 (5.4)	5 (10.4)	15 (15.5) ^*^	23 (8.0)
	*KatG* + *inhA*	AGC → ACC + C → T	*Ser*315*Thr + C*(−15) → *T*	0 (0.0)	0 (0.0)	0 (0.0)	0 (0.0)	0 (0.0)	3 (3.1)	3 (1.0)
	Other locus	Other mutations	Other substitutions	6 (15.0)	40 (88.9) ^*^	10 (47.6) ^*^	0 (0.0) ^*^	0 (0.0) ^*^	2 (2.1) ^*^	58 (20.1)
Total				40 (100.0)	45 (100.0)	21 (100.0)	37 (100.0)	48 (100.0)	97 (100.0)	288 (100.0)
RIF										
	*rpoB*	CTG → CCG	*Leu*511*Pro*	8 (17.0)	1 (1.8) ^*^	1 (2.6) ^*^	3 (6.4)	4 (5.8)	10 (8.1)	27 (7.1)
		CAA → CCA	*Gln*513*Pro*	0 (0.0)	0 (0.0)	0 (0.0)	0 (0.0)	0 (0.0)	1 (0.8)	1 (0.3)
		CAA → AAA	*Gln*513*Lys*	2 (4.3)	0 (0.0)	1 (2.6)	1 (2.1)	1 (1.5)	0 (0.0)	5 (1.3)
		GAC → GGC	*Asp*516*Gly*	0 (0.0)	1 (1.8)	0 (0.0)	0 (0.0)	1 (1.5)	2 (1.6)	4 (1.0)
		GAC → GTC	*Asp*516*Val*	4 (8.5)	1 (1.8)	0 (0.0)	2 (4.3)	2 (2.9)	3 (2.4)	12 (3.1)
		GAC → TAC	*Asp*516*Tyr*	0 (0.0)	0 (0.0)	0 (0.0)	2 (4.3)	1 (1.5)	2 (1.6)	5 (1.3)
		CAC → CGC	*His*526*Arg*	1 (2.1)	0 (0.0) ^*^	1 (2.6)	1 (2.1)	4 (5.8)	1 (0.8)	8 (2.1)
		CAC → CTC	*His*526*Leu*	1 (2.1)	0 (0.0)	1 (2.6)	1 (2.1)	3 (4.4)	1 (0.8)	7 (1.8)
		CAC → GAC	*His*526*Asp*	4 (8.5)	1 (1.8)	0 (0.0)	4 (8.5)	4 (5.8)	10 (8.1)	23 (6.0)
		CAC → TAC	*His*526*Tyr*	3 (6.4)	1 (1.8)	1 (2.6)	5 (10.6)	9 (13.0)	13 (10.5)	32 (8.4)
		TCG → TGG	*Ser*531*Trp*	2 (4.3)	1 (1.8) ^*^	0 (0.0)	1 (2.1) ^*^	3 (4.4) ^*^	1 (0.8) ^*^	8 (2.1)
		TCG → TTG	*Ser*531*Leu*	12 (25.5)	5 (8.8)	14 (35.9)	25 (53.2)	34 (49.3)	69 (55.6)	159 (41.5)
		CTG → CCG	*Leu*533*Pro*	0 (0.0)	1 (1.8)	0 (0.0)	0 (0.0)	2 (2.9)	5 (4.0)	8 (2.1)
		CTG → CCG+ GAC → GGC	*Leu*511*Pro* + *Asp*516*Gly*	1 (2.1)	0 (0.0)	0 (0.0)	1 (2.1)	0 (0.0)	0 (0.0)	2 (0.5)
		CTG → CCG + GAC → TAC	*Leu*511*Pro* + *Asp*516*Tyr*	1 (2.1)	0 (0.0)	0 (0.0)	0 (0.0)	0 (0.0)	0 (0.0)	1 (0.3)
		CTG → CCG + GAC → GTC	*Leu*511*Pro* + *Asp*516*Val*	0 (0.0)	0 (0.0)	0 (0.0)	0 (0.0)	0 (0.0)	1 (0.8)	1 (0.3)
		CTG → CCG + CAC → TAC	*Leu*511*Pro* + *His*526*Tyr*	0 (0.0)	0 (0.0)	0 (0.0)	0 (0.0)	0 (0.0)	2 (1.6)	2 (0.5)
		CAA → CCA + GAC → GTC	*Gln*513*Pro* + *Asp*516*Val*	0 (0.0)	0 (0.0)	0 (0.0)	0 (0.0)	0 (0.0)	1 (0.8)	1 (0.3)
		GAC → GGC + CTG → CCG	*Asp*516*Gly* + *Leu*533*Pro*	2 (4.3)	0 (0.0)	0 (0.0)	0 (0.0)	0 (0.0)	1 (0.8)	3 (0.8)
	Other locus	Other mutations	Other substitutions	6 (12.8)	45 (79.0) ^*^	20 (51.3) ^*^	1 (2.1)	1 (1.5) ^*^	1 (0.8) ^*^	74 (19.3)
Total				47 (100.0)	57 (100.0)	39 (100.0)	47 (100.0)	69 (100.0)	124 (100.0)	383 (100.0)
MDR	*rpoB*+ *KatG*	CTG → CCG+ AGC → ACC	*Leu*511*Pro* + *Ser*315*Thr*	8 (21.6)	0 (0.0) ^*^	0 (0.0) ^*^	0 (0.0) ^*^	3 (7.0)	6 (8.1)	17 (7.0)
		GAC → GGC + AGC → AAC	*Asp*516*Gly* + *Ser*315*Asn*	0 (0.0)	0 (0.0)	0 (0.0)	0 (0.0)	0 (0.0)	1 (1.4)	1 (0.4)
		GAC → GGC + AGC → ACC	*Asp*516*Gly* + *Ser*315*Thr*	0 (0.0)	0 (0.0)	0 (0.0)	0 (0.0)	0 (0.0)	1 (1.4)	1 (0.4)
		GAC → GTC + AGC → ACC	*Asp*516*Val* + *Ser*315*Thr*	3 (8.1)	0 (0.0)	0 (0.0)	1 (3.5)	2 (4.7)	1 (1.4)	7 (2.9)
		GAC → TAC + AGC → ACC	*Asp*516*Tyr* + *Ser*315*Thr*	0 (0.0)	0 (0.0)	0 (0.0)	1 (3.5)	1 (2.3)	1 (1.4)	3 (1.2)
		CAC → CGC + AGC → ACC	*His*526*Arg* + *Ser*315*Thr*	1 (2.7)	0 (0.0) ^*^	1 (5.0)	1 (3.5)	3 (7.0)	0 (0.0)	6 (2.5)
		CAC → CTC + AGC → ACC	*His*526*Leu* + *Ser*315*Thr*	1 (2.7)	0 (0.0)	0 (0.0)	1 (3.5)	0 (0.0)	0 (0.0)	2 (0.8)
		CAC → GAC + AGC → AAC	*His*526*Asp* + *Ser*315*Asn*	1 (2.7)	0 (0.0)	0 (0.0)	0 (0.0)	0 (0.0)	0 (0.0)	1 (0.4)
		CAC → GAC + AGC → ACC	*His*526*Asp* + *Ser*315*Thr*	3 (8.1)	1 (2.6)	0 (0.0)	3 (10.3)	3 (7.0)	5 (6.8)	15 (6.2)
		CAC → TAC + AGC → ACC	*His*526*Tyr* + *Ser*315*Thr*	1 (2.7)	0 (0.0)	1 (5.0)	5 (17.2)	5 (11.6)	7 (9.5)	19 (7.9)
		TCG → TGG + AGC → AAC	*Ser*531*Trp* + *Ser*315*Asn*	0 (0.0)	0 (0.0) ^*^	0 (0.0)	0 (0.0)	1 (2.3) ^*^	0 (0.0) ^*^	1 (0.4)
		TCG → TGG + AGC → ACC	*Ser*531*Trp* + *Ser*315*Thr*	2 (5.4)	1 (2.6)	0 (0.0)	0 (0.0)	1 (2.3)	1 (1.4)	5 (2.1)
		TCG → TTG + AGC → AAC	*Ser*531*Leu* + *Ser*315*Asn*	0 (0.0)	0 (0.0)	0 (0.0)	0 (0.0)	2 (4.7)	4 (5.4)	6 (2.5)
		TCG → TTG + AGC → ACC	*Ser*531*Leu* + *Ser*315*Thr*	8 (21.6)	2 (5.1)	8 (40.0)	14 (48.3)	18 (41.9)	34 (46.0)	84 (34.7)
		CTG → CCG + AGC → ACC	*Leu*533*Pro* + *Ser*315*Thr*	0 (0.0)	1 (2.6)	0 (0.0)	0 (0.0)	0 (0.0)	3 (4.1)	4 (1.7)
	*rpoB* + *inhA*	GAC → GGC + C → T	*Asp*516*Gly* + *C*(−15) → *T*	0 (0.0)	0 (0.0)	0 (0.0)	0 (0.0)	1 (2.3)	0 (0.0)	1 (0.4)
		CAC → CGC + C → T	*His*526*Arg* + *C*(−15) → *T*	0 (0.0)	0 (0.0)	0 (0.0)	0 (0.0)	1 (2.3)	0 (0.0)	1 (0.4)
		TCG → TGG + C → T	*Ser*531*Trp* + *C*(−15) → *T*	0 (0.0)	0 (0.0)	0 (0.0)	0 (0.0)	1 (2.3)	0 (0.0)	1 (0.4)
		TCG → TTG + C → T	*Ser*531*Leu* + *C*(−15) → *T*	0 (0.0)	0 (0.0)	1 (5.0)	2 (6.9)	1 (2.3)	1 (1.4)	5 (2.1)
	*rpoB* + *rpoB* + *KatG*	CTG → CCG + GAC → GGC + AGC → ACC	*Leu*511*Pro* + *Asp*516*Gly + Ser*315*Thr*	1 (2.7)	0 (0.0)	0 (0.0)	1 (3.5)	0 (0.0)	0 (0.0)	2 (0.8)
		CTG → CCG + GAC → GTC + AGC → ACC	*Leu*511*Pro* + *Asp*516*Val* + *Ser*315*Thr*	0 (0.0)	0 (0.0)	0 (0.0)	0 (0.0)	0 (0.0)	1 (1.4)	1 (0.4)
		CTG → CCG + CAC → TAC + AGC → ACC	*Leu*511*Pro* + *His*526*Tyr* + *Ser*315*Thr*	0 (0.0)	0 (0.0)	0 (0.0)	0 (0.0)	0 (0.0)	1 (1.4)	1 (0.4)
		CAA → CCA + GAC → GTC + AGC → ACC	*Gln*513*Pro* + *Asp*516*Val* + *Ser*315*Thr*	0 (0.0)	0 (0.0)	0 (0.0)	0 (0.0)	0 (0.0)	1 (1.4)	1 (0.4)
		GAC → GGC + CTG → CCG + AGC → AAC	*Asp*516*Gly* + *Leu*533*Pro* + *Ser*315*Asn*	2 (5.4)	0 (0.0)	0 (0.0)	0 (0.0)	0 (0.0)	0 (0.0)	2 (0.8)
		GAC → GGC + CTG → CCG + AGC → ACC	*Asp*516*Gly* + *Leu*533*Pro* + *Ser*315*Thr*	0 (0.0)	0 (0.0)	0 (0.0)	0 (0.0)	0 (0.0)	1 (1.4)	1 (0.4)
	*rpoB* + *KatG* + *inhA*	TCG → TTG + AGC → ACC + C → T	*Ser*531*Leu* + *Ser*315*Thr* + *C*(−15) → *T*	0 (0.0)	0 (0.0)	0 (0.0)	0 (0.0)	0 (0.0)	3 (4.1)	3 (1.2)
	Other locus	Other mutations	Other substitutions	6 (16.2)	34 (87.2) ^*^	9 (45.0) ^*^	0 (0.0) ^*^	0 (0.0) ^*^	2 (2.8) ^*^	51 (21.1)
	Total			37 (100.0)	39 (100.0)	20 (100.0)	29 (100.0)	43 (100.0)	74 (100.0)	242 (100.0)

Note: ^*^ indicates that the difference is statistically significant compared with 2014

In the isoniazid resistance mutation sites, *katG* and promoter *inhA* were 70.8% (204/288) and 8.0% (23/288), and combinatorial mutation of *katG* + *inhA* was 1.0% (3/288), respectively. *katG* was the most common mutation, and its mutation was all higher than 79.0% from 2017 to 2019. In 2019, the mutation rate of *inhA* was significantly increased (15.5%, 15/97, *P* = 0.006). In addition, the combinatorial mutation of *katG* + *inhA* was only found in 2019.

Among rifampin resistance mutation sites, *rpoB*531 (43.6%, 167/383), *rpoB*526 (18.3, 70/383) and *rpoB*511 (7.0, 27/383) were the most frequent. The mutation rates of *rpoB*531isolates from 2017 to 2019 were significantly higher than those from 2014 (*P* < 0.05).

The mutation rates of *rpoB* + *inhA* and *rpoB* + *katG* in MDR-TB strains were 3.3% (8/242). The *rpoB* + *katG*+ *inhA* mutation was only found in three isolates in 2019.While the *rpoB* + *katG* mutation rate was 71.1% (172 / 242), *rpoB*531 + *katG*315 mutation in MDR-TB isolates was significantly higher in 2018 (51.2%, 22/43, *P* = 0.028) and 2019 (52.7%, 39/74, *P* = 0.010) than in 2014 (7.7%, 3 / 39).

This study also compared the mutation sites of drug-resistant strains from 2014 to 2019, and some new drug-resistant gene combinations were detected. For isoniazid, combinatorial mutation of Ser315 → Thr + inhAT_15 emerged. For rifampin, combinatorial mutation of Gln513 → Pro, Leu511 → Pro + Asp516 → Val appeared. For MDR-TB strains, Leu511 → Pro, Asp516 → Val + Ser315 → Thr were also found, and the drug-resistant mutation sites were constantly diversified.

## Discussion

In 2017, the Southeast Asia and the Western Pacific region saw the largest number of new TB cases, accounting for 62% of all new cases worldwide. China is one of the three countries with the highest drug-resistant TB in the world. Previous studies have shown that the clinical and molecular characteristics of drug-resistant strains in China vary from region to region [8–16]. Hainan is the only tropical island in China. Its unique tropical climate and relatively low population mobility may affect the infection and drug resistance of tuberculosis. Determining the change in the TB drug-resistance rate over time and its current status in Hainan are essential to adequately administer anti-TB regimens and achieve successful treatment. This study was a large population and long-term-based retrospective study conducted in Hainan Province, China. To our knowledge, this is the first study providing comprehensive assessment of the dynamic changes of drug resistance rate and the mutation sites of isoniazid and rifampin resistance in Hainan Province.

The overall percentage of retreatment cases was 44.4%, while, a review showed that the median percentage of TB patients experiencing an episode of retreatment TB after treatment completion was 3.4% (interquartile range [IQR] 1.6–6.0, range 0.4–16.7) [17],suggesting that the retreatment cases is still a big challenge in controlling the TB epidemic in Hainan.

The present study showed that 44.3% (658/1484) of patients had drug-resistant disease, meanwhile the proportions of MDR- and XDR-TB among patients were 24.9 and 2.2%, nearly two times the proportions presented in the data from a China Clinical Tuberculosis Centre in 2017 [18]. According to a WHO report, the global MDR/RR-TB (multidrug resistant tuberculosis / rifampin resistant tuberculosis) rate was 3.3% (95%CI: 2.4–4.4%) for new cases and 18% (95%CI: 9.7–27%) for retreatment cases in 2019, while in China, the MDR/RR-TB rate was 7.1% (95%CI: 5.6–8.7%) for new cases and 23% (95%CI: 23–24%) for retreatment cases in the same year. The drug-resistant TB prevalence was significantly different in china different regions. The Eastern coastal region is the most developed economic region with the lowest total drug-resistant TB prevalence (any drug resistance: 28%; 95% CI 25–32%; MDR: 9%; 95% CI, 8–12%) and the lowest number of new cases (any drug resistance: 21%; 95% CI, 19–23%; MDR: 4%; 95% CI, 3–5%). The Northwest is the least developed area with the lowest drug-resistant TB prevalence for retreatment cases (any drug resistance: 45%; 95% CI, 36–55%; MDR: 17%; 95% CI, 11–26%). Overall, the drug-resistant TB in China is notably severe and shows regional epidemiologic characteristic [19, 20]. However, our data showed that, in 2019, the MDR/RR-TB rate was11.4% for new cases and 55.7% for retreatment cases in Hainan, which was significantly higher than the average rate of both the global and China’s. It showed a serious epidemic of drug-resistant tuberculosis in Hainan. The high TB drug resistance rate might be partially due to low economic level and TB management in Hainan. It is difficult for a low household income family to cover high drugs resistant treatment costs, which leads to a poor adherence to MDR-TB or XDR-TB treatment, and causes the emergence of drug resistance. In addition, many drugs resistant patients might not have access to adequate treatment of sufficient quality.

The changes of drug resistance rate of new cases were not statistically significant. However, the resistance rates of first-line anti-tuberculosis drugs and second-line anti-tuberculosis drugs increased significantly in retreatment patients with rifampin resistant TB increased from 45.9% in 2014 to 60.3% in 2019 and protionamide increased at an annual rate of0.18%. A higher risk of drug resistance was found among retreatment patients, similar results could be found in other reports [21, 22]. This implies that acquired-drug resistance may play an increasing role in the DR-TB epidemic in Hainan. Hence, some appropriate strategies must be implemented to increase continuity of treatment and reduce the rate of treatment default.

We also found that people older than 64 years of age had a lower risk of any drug-resistant TB, MDR-TB and XDR-TB. This is consistent with the conclusion of a systematic review of European studies which concluded that MDR-TB cases are more likely to occur in patients younger than 65 years of age [23, 24]. The higher risk of getting MDR-TB in people under 65 years may be attributed to the use of RIF for anti-TB treatment from around 1965. TB cases in older patients are usually considered as the infecting strains may be more ancient, and carry a lower risk of becoming resistant to drug, the frequency of DR-TB peaked in young adulthood and the age profile of DR-TB agreed with other reports [20].

Several recent studies have examined the contribution of *katG* and *inhA* promoter mutations in drug-resistant TB isolates, and the results revealed significant geographic diversity across regions [25–27]. This study found that the most common mutation of MDR-TB was 34.7% (Ser531 → Leu + Ser315 → Thr), however, a study in Brazil showed that was 41.7% [28]. In our study, the mutation rate in *katG*315 was 71.9%, which was higher than the mutation rates reported in Poland (66.0%) and Hebei Province, China (69.9%) [29, 30]. The most common mutation of rifampin was *rpoB*531 (43.6%, 167/383), which was lower than the result from Kyrgyz Republic (64.8%) [31]. The regional differences in the frequencies of mutations associated with resistance may reflect the diversity in molecular characteristics of DR-TB isolates circulating in geographically distinct areas, and provide insights for the development of molecular-based diagnostic tests.

Another interesting finding was that a combined mutation of *katG* + *inhA*, which was rarely reported before, was identified in the study. Moreover, a simultaneous mutation in *rpoB* + *katG* + *inhA* was also identified, indicating that *M. tuberculosis* strains were constantly mutating. These data might be helpful in the design and development of new anti-TB drugs. There were still some resistant isolates harboring no mutation within the sequenced regions. This implied that these isolates probably harbored mutations outside the sequenced area or that the resistance may be caused by other mechanisms, such as efflux pumps [32].

Due to the limitations of retrospective data collection, the education background, socioeconomic status, and living conditions of the patients involved in this study were not well described and recorded. The interplay of these factors and how could it affect the epidemic of drug-resistant TB are somewhat neglected. Well-designed studies with comprehensive and detailed research data in China should thus be conducted in the future.

## Conclusions

Despite these limitations, the trends of different drug resistance patterns overtime were examined and a better understanding of the epidemic characteristics of TB cases in Hainan was obtained. First, the drug-resistant TB rate remains high throughout the study. Second, the age and treatment history were independent risk factors of TB drug resistance. Third, different mutation rates and patterns are identified.

## Materials and methods

### Study population and data collection

This study was carried out from January 2014 to December 2019 at the Second Affiliated Hospital of Hainan Medical University, which serves as the sole specialized TB hospital in Hainan. Information for all patients (age, gender, TB contact history, and TB treatment history, etc.) was collected and recorded. Only one isolate per patient was collected and tested.

### Laboratory pretreatment

Pulmonary samples were collected by expectoration, gastric aspiration, and sputum induction. Extra pulmonary samples (pleural fluid, spinal fluid, and lymph nodes) were collected by pleural tap, lumbar puncture, lymph node biopsy, fine needle aspiration, and other techniques. The patients’ samples were placed in a microcentrifuge tube, and processed for smear and culture. To identify the presence of acid-fast bacilli, we used *Ziehl-Neelsen* staining (Baso, Zhuhai, China) for smear microscopy. Each sample was inoculated into the acidic modified Lowenstein-Jensen (Cell Biotech Co., Ltd., Hainan, China) culture medium. Strain isolation and identification were performed in a tuberculosis reference laboratory of the Second Affiliated Hospital of Hainan Medical University. All operations strictly comply with standard biosecurity and institutional safety procedures.

### Drug sensitivity test

Following cultivation, the *M. tuberculosis* was assessed for drug sensitivity using a Lowenstein-Jensen culture medium and the following drug concentrations: isoniazid (0.2 μg/mL), rifampin (40.0 μg/mL), ethambutol (2.0 μg /mL), streptomycin (4.0 μg/mL), capreomycin (2.0 μg/mL), kanamycin (40.0 μg/mL), ofloxacin (30.0 μg/mL), and protionamide (40.0 μg/mL). Isolates with growth proportion for > 1% on medium containing anti-TB drugs compared with the growth on drug free medium were resistant to those drugs [13].

### Detection by CapitalBio™ DNA microarray

This study was based on *M. tuberculosis* drug resistance gene detection kit (CapitalBio™ DNA microarray method, Beijing CapitalBio Technology, 301,035), which can specifically detect the mutations of *rpoB*, *katG* and *inhA*. Laboratory operations were performed according to the manufacturer’s instruction [33]. For *rpoB* gene six loci were detected, including 531 TCG → TTG, 531 TCG → TGG, 526 CAC → GAC, 526 CAC → TAC, 526 CAC → CTC, 526 CAC → CGC, 511 CTG → CCG, 513 CAA → CCA, 513 CAA → AAA, 516 GAC → GTC, 516 GAC → GGC and 533 CTG → CCG. For *katG* gene one locus was detected, which was 315 AGC → ACC and 315 AGC → AAC. For the promoter of *inhA* gene, one locus was detected, which was − 15 C → T.

### Statistical analysis

The Chi-square test or the Fisher’s exact test were chosen to assess the difference between different groups. Univariate and multivariate analysis were used to evaluate the influencing factors related to drug resistance of tuberculosis by SPSS 21.0 software as well. A two-tailed *P* < 0.05 was considered statistically significant.

## Data Availability

The data that support the findings of this study are available from the Second Affiliated Hospital of Hainan Medical University but restrictions apply to the availability of these data, which were used under license for the current study, and so are not publicly available. Data are however available from the authors upon reasonable request and with permission of the Second Affiliated Hospital of Hainan Medical University.

## Abbreviations

CPM: Capreomycin
DR-TB: Drug-resistant tuberculosis
EMB: Ethambutol
INH: Isoniazid
KAR: Kanamycin
MDR-TB: Multidrug-resistant tuberculosis
MDR: Multi-drug resistant
*M. tuberculosis*: *Mycobacterium tuberculosis*
PTO: Protionamide
RIF: Rifampin
STR: Streptomycin
TB: Tuberculosis
XDR-TB: Extensively drug-resistant tuberculosis
XDR: Extensively drug-resistant
